# Procalcitonin and biomarkers for stroke-associated pneumonia: a systematic review and meta-analysis

**DOI:** 10.1186/s12890-025-03750-6

**Published:** 2025-06-09

**Authors:** Yuan Wang, Tingting Wang, Shouqin Hu, Yuntao Cheng, Chongyue Du, Guolong Xu

**Affiliations:** Department of Emergency, Jiashan County Traditional Chinese Medicine Hospital, No. 38 Gujiadai, Weitang Street, Jiashan County, Jiaxing, Zhejiang Province 314100 China

**Keywords:** Stroke, Pneumonia, Procalcitonin, Biomarkers, Clinical prediction model

## Abstract

**Background:**

Stroke-associated pneumonia (SAP) is a common and severe complication following stroke, significantly impacting recovery and outcomes. Early identification of biomarkers and development of predictive models are essential for SAP diagnosis and prevention. This study systematically evaluated the diagnostic value of procalcitonin (PCT) and other biomarkers for SAP and explored their integration into predictive models.

**Methods:**

A systematic review and meta-analysis were conducted by searching PubMed, Web of Science, and CNKI databases for studies published up to March 2023. Inclusion criteria focused on studies reporting biomarkers for SAP diagnosis and predictive models. Statistical analyses included pooled sensitivity, specificity, diagnostic odds ratio (DOR), and area under the receiver operating characteristic curve (AUC) using RevMan 5.4 and R software.

**Results:**

This meta-analysis included 11 studies with 1,478 patients and found that PCT levels were significantly elevated in SAP patients, particularly those with ischemic stroke (standardized mean difference [SMD] = 2.89, 95% confidence interval [CI] = 1.74–4.04). PCT demonstrated high diagnostic accuracy, with a pooled sensitivity of 0.84, specificity of 0.89, DOR of 48.78, and AUC of 0.91, outperforming other biomarkers like CRP and IL-6. Predictive models incorporating biomarkers improved risk stratification, though heterogeneity among studies underscores the need for standardization.

**Conclusions:**

PCT is a reliable biomarker for SAP diagnosis, offering high sensitivity and specificity. Combining PCT with predictive models can enhance risk assessment and early detection of SAP. Further research is necessary to refine prediction models and validate the clinical application of biomarkers across diverse populations. This study underscores the importance of biomarkers in guiding SAP prevention and management strategies.

**Supplementary Information:**

The online version contains supplementary material available at 10.1186/s12890-025-03750-6.

## Background

Stroke is a leading cause of disability and death, imposing a significant economic burden on individuals, families, and society [1]. Various complications, including seizures, gastrointestinal bleeding, pressure sores, and infections often accompany stroke. Among these, acute stroke-related infections are frequent, exacerbating the severity of the disease, with lung and urinary tract infections (UTIs) being particularly common [2]. In recent years, researchers have increasingly focused on Stroke-Associated Pneumonia (SAP). In 2003, Hilker et al. first defined SAP [3]. Subsequently, under the organization of Smith et al., European experts developed a comprehensive consensus on the definition and diagnosis of SAP in 2015. This consensus defined SAP as pneumonia that occurs within seven days after stroke onset in non-ventilated patients [4]. The incidence of SAP is approximately 14% [5]. The neutrophil-to-lymphocyte ratio (NLR) is strongly associated with SAP in patients with acute ischemic stroke (AIS). Studies indicate that an elevated NLR can predict SAP occurrence, facilitating early identification of high-risk patients and providing potential insights for prophylactic antibiotic therapy [6]. Building on this, Buonacera et al. further investigated the association between NLR and SAP in elderly patients aged 75 and older, assessing its validity as a prognostic tool for community-acquired pneumonia in this population [7, 8]. Additionally, various prognostic markers, including NLR, PLR, MLR, PNI, SIRI, SII, GPS, mGPS, and PI, have been identified as predictors of SAP, with NLR recognized as the most reliable predictor for early SAP detection [9]. SAP is not merely a combination of “stroke” and “pneumonia”; its complex pathophysiological mechanisms pose significant challenges for clinical diagnosis and treatment.

**Fig. 1:**
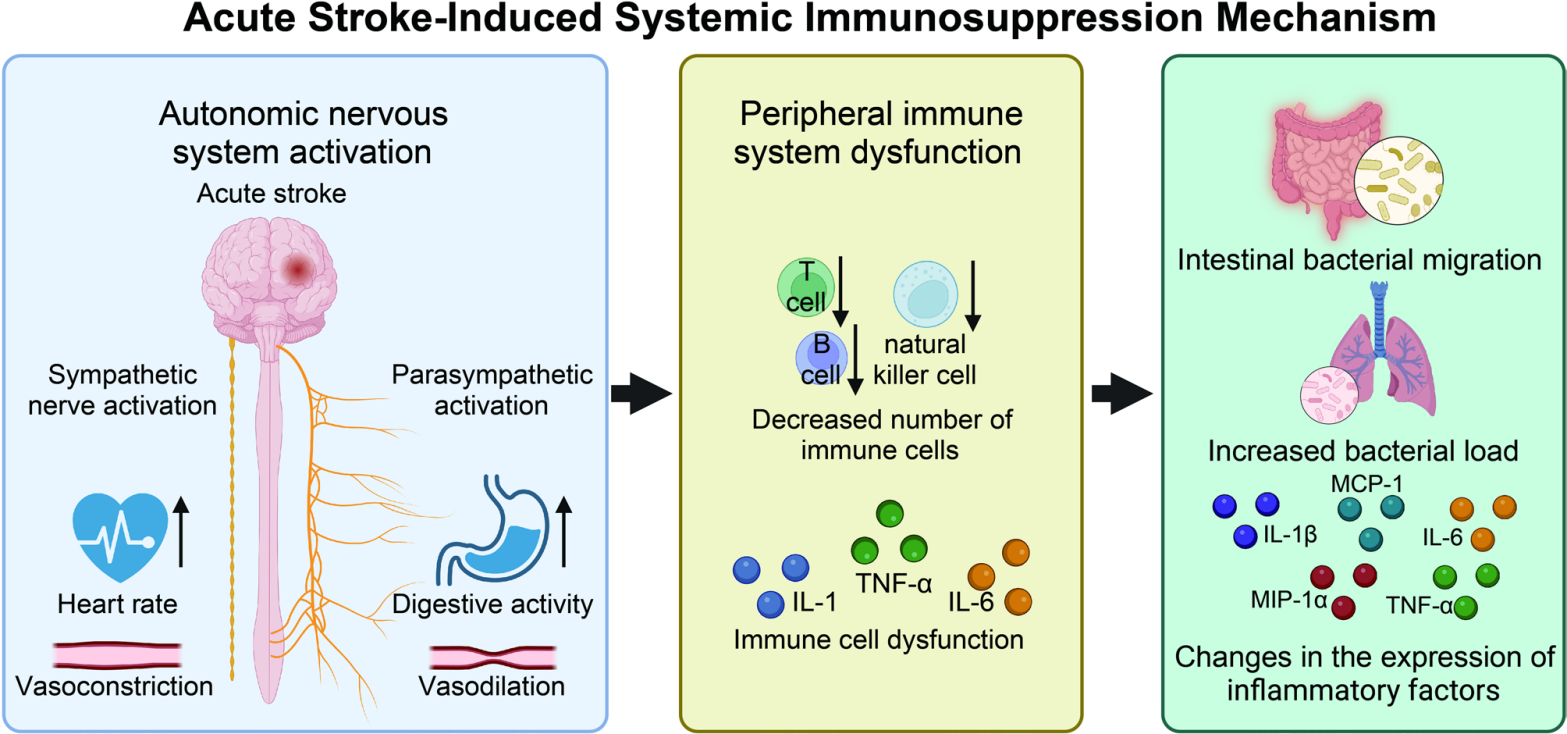
Systemic immunosuppression (SIS) induced by acute stroke

Acute stroke can induce systemic immunosuppression (SIS) (Fig. 1). Excessive activation of the autonomic nervous system serves to limit central immune responses and prevent additional brain cell damage through stroke-induced immunosuppression. This post-stroke peripheral immunosuppression hampers the normal bactericidal immune function, making the host more susceptible to infection [10, 11]. Studies have shown that the peripheral immune system function of stroke patients is significantly impaired, including reduced immune cell counts, altered cytokine levels, and immune cell dysfunction. In hemorrhagic stroke, mouse models display significant immunosuppression at different time points, particularly on the third day, with some improvement by the seventh day. Additionally, cytokine expression levels in the lung tissue of mice change following hemorrhagic stroke. On the seventh day post-stroke, bacterial load in lung tissue significantly increases, and the expression levels of inflammatory cytokines such as Interleukin-1 beta (IL-1β), Interleukin-6 (IL-6), MCP-1, MIP-1α, and Tumor Necrosis Factor-alpha (TNF-α) show significant changes, indicating immune system dysregulation and sustained inflammation. Furthermore, hemorrhagic stroke damages the intestinal barrier function in mice, leading to villous atrophy, blurred structure, reduced goblet cell count, and increased intestinal permeability. These damages facilitate the translocation of gut bacteria to other tissues and organs, such as the lungs, thereby increasing the risk of systemic infection [12]. Using the MCAO model, researchers found that gut bacteria can migrate to the lungs, liver, and spleen, indicating significant changes in the gut microbiota post-stroke. Early brain injury induced by stroke triggers systemic inflammatory response syndrome (SIRS) and the release of systemic inflammatory mediators. The systemic inflammatory response peaks within six hours post-stroke. To control excessive inflammation and immune response, SIS emerges as a compensatory mechanism. However, excessive immunosuppression increases the likelihood of infection, thereby affecting the recovery and mortality rates of stroke patients and experimental animals [13].

The sources of potential pathogens in SAP include colonizing bacteria in the oral cavity and respiratory tract, persistent bacteria from community or hospital environments, aspirated foreign materials due to dysphagia, and bacterial translocation from the host’s gut microbiota [14–16]. Clinical studies have identified several risk factors for SAP, including severe neurological impairment, posterior circulation infarction, advanced age, impaired consciousness, and dysphagia. Additionally, atrial fibrillation, hypertension, and diabetes are significant risk factors [17, 18]. SAP progresses rapidly and is severe, prolonging hospital stays and increasing the risk of poor prognosis and mortality in stroke patients [19]. Therefore, it is crucial to accurately assess the condition of stroke patients, predict the occurrence of SAP, and implement effective prevention and treatment strategies in clinical practice.

## Methods

### Meta-analysis literature search strategy

To comprehensively explore and evaluate the clinical and inflammatory biomarkers of SAP, we devised a detailed literature search strategy. This plan was implemented across major medical databases, including PubMed, Web of Science, and CNKI, covering all relevant literature from the inception of each database up to July 2024. We used a series of carefully selected keywords and expressions to guide our search process, aiming to maximize the coverage of studies related to SAP clinical and inflammatory biomarkers. The search keywords and expressions included, but were not limited to, the following: “Stroke-Associated Pneumonia,” “SAP,” “Clinical Biomarkers of SAP,” “Inflammatory Biomarkers of SAP,” “CRP,” “Procalcitonin,” “IL-6,” and “Biomarkers in Stroke.” Additionally, specific biomarkers and related terms such as “C-reactive protein,” “PCT,” and “interleukin-6” were included in the search vocabulary.

During the literature search, we adjusted our strategy according to the characteristics of each database to ensure the results were both comprehensive and high-quality. To further broaden the scope and depth of our search, we also performed manual searches, including reviewing the reference lists of relevant journals and important conference records. This systematic review and meta-analysis was conducted in accordance with the Preferred Reporting Items for Systematic Reviews and Meta-Analyses (PRISMA) guidelines.

### Inclusion and exclusion criteria

To ensure the quality and specificity of this systematic review and meta-analysis, we carefully selected studies that met specific criteria. We focused particularly on randomized controlled trials (RCTs) involving adult patients with SAP. Included studies were required to emphasize clinical and inflammatory biomarkers, clinical prediction models, and preventive strategies for SAP while also ensuring that the studies provided objective and reliable outcome measures, such as assessments using C-reactive protein (CRP), procalcitonin (PCT), and IL-6.

For exclusion criteria, we eliminated studies that did not meet these standards, including those involving non-adult subjects, non-human studies, studies on other types of pneumonia or non-stroke-related diseases, incomplete or unidentifiable data, and non-RCTs, cohort studies, reviews, case reports, and expert opinions. Additionally, duplicate studies were excluded to avoid redundancy and bias in the results. This approach aimed to eliminate studies that could affect the accuracy and reliability of the meta-analysis, ensuring the quality and consistency of the selected literature.

### Literature coding and quality assessment

Each study was meticulously coded to include essential information such as the author, publication year, sample size, and key data on SAP-related clinical and inflammatory biomarkers, clinical prediction models, and prevention strategies. We used Excel for data organization, ensuring consistency and accuracy throughout the coding process. The quality assessment of the literature followed the Cochrane Risk of Bias Tool to ensure the reliability of the study results. Specifically, we evaluated each study’s random sequence generation, allocation concealment, blinding, data integrity, selective reporting, and other potential biases. This systematic quality assessment aimed to identify high-quality research, providing a solid data foundation for our meta-analysis.

### Data extraction

In this Meta-analysis, the data extraction process focuses on collecting detailed information about each study related to SAP. This includes specific records of the biomarkers involved in each study (such as CRP, PCT, IL-6) and the clinical prediction models used (e.g., multivariable logistic regression models). Additionally, we will record key statistical data such as sample sizes, experimental group settings, p-values, and confidence intervals. This step is crucial for subsequent statistical analysis, as it not only helps us compare and analyze the effectiveness of different clinical strategies in the prevention and treatment of SAP but also allows us to assess the reliability and statistical significance of the study results. By employing a systematic and meticulous data extraction method, we aim to ensure that the results of the Meta-analysis are highly accurate and reliable, thus providing a solid scientific basis for optimizing SAP treatment.

### Statistical methods

In this study, we used RevMan 5.4 and R software to conduct a meta-analysis, selecting effect sizes and their 95% confidence intervals (CIs) as the primary metrics. To assess heterogeneity among the included studies, we employed Cochran’s Q test and the I² statistic. Sensitivity analysis was conducted to evaluate the robustness of the results. Additionally, publication bias was assessed using funnel plots and Egger’s test. All statistical tests were conducted at a significance level of *p* < 0.05. These statistical methods allowed us to comprehensively evaluate the efficacy and safety of SAP prevention and treatment measures, providing reliable evidence for clinical practice.

## Results

### Literature screening summary and quality assessment of included studies

**Fig. 2:**
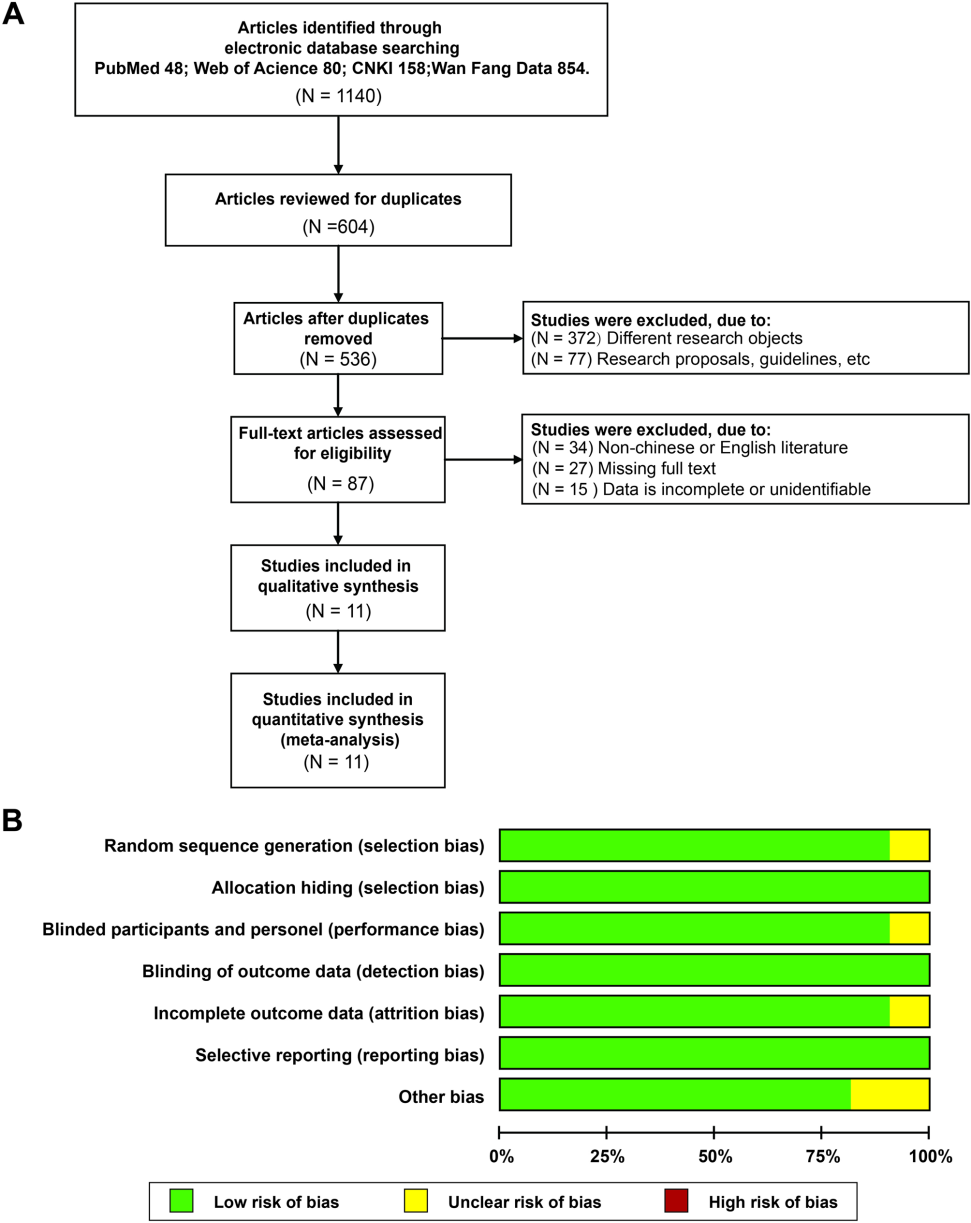
Literature selection process and quality assessment results. Note: (**A**) Flowchart of literature inclusion; (**B**) Summary of bias risk assessment for included studies

A total of 1,140 studies were identified through searches of PubMed, Web of Science, CNKI, and Wan Fang Data. After removing duplicates, 536 articles remained. Based on title and abstract screening, 449 articles were excluded due to being non-randomized or unrelated to stroke. The full texts of the remaining 87 articles were reviewed, and 76 were excluded due to missing data or insufficient information. Ultimately, 11 studies met the inclusion criteria and were included in the quantitative meta-analysis (Fig. 2A; Table 1).

**Table 1:** Information about the literature that met the inclusion criteria

First Author	Publication	Sample type	Sample size	Age of subject	Detection	Treatment	Case Presentation	Diagnosis
Wang Zeshuai [25]	2024	Ischemic	176	65–85	IFA	Not specified	Studied PCT levels in relation to SAP	Significantly higher PCT levels in SAP patients
Gu Honghong [26]	2023	Ischemic	178	60–80	CLIA	Not specified	Examined sCD163 and PCT levels in diagnosing SAP	Higher sCD163 and PCT levels indicate SAP
Wang Xiaobin [27]	2023	Ischemic	150	65–85	ELISA	Not specified	Evaluated serum MMP-9 levels and SAP correlation	Elevated MMP-9 correlated with SAP
Tang Xiaobo [28]	2022	Ischemic	160	60–80	IFA	Not specified	Investigated CGRP and MMP-9 in SAP diagnosis	Elevated CGRP and MMP-9 levels in SAP diagnosis
Li Min [29]	2022	Hemorrhagic	180	≥ 60	IFA	Not specified	Assessed infection markers in elderly hypertensive patients with SAP	Significantly higher infection markers in SAP patients
Hu Chaosheng [30]	2022	Ischemic	155	≥ 60	CLIA	Not specified	Explored PCT, CRP, and CRP/Alb ratios for SAP early diagnosis	Higher PCT, CRP, and CRP/Alb levels predict SAP
Zhao Lu [31]	2021	Ischemic	83	61.3 ± 6.5	IFA	Antibiotic treatment based on PCT levels	Acute stroke within 24 h of onset	Serum PCT and HLA-DR levels measured; SAP diagnosed within 7 days
Xu Mojun [32]	2020	Ischemic	150	67.3 ± 8.1	CLIA	Antibiotic treatment based on PCT and CRP/Alb ratio	Acute ischemic stroke within 24 h of onset	Serum PCT, CRP/Alb ratio measured; SAP diagnosed within 7 days
Li Hua [33]	2020	Ischemic	100	65.5 ± 7.2	ELISA	Antibiotic treatment based on HBP levels	Acute stroke within 48 h of onset	Serum HBP levels measured; SAP diagnosed within 7 days
Zhang Huaping [34]	2020	Hemorrhagic	165	55–80	IFA	Not specified	Determined diagnostic values of NLR, sTREM-1, and PCT in SAP	Higher NLR, sTREM-1, and PCT levels in SAP diagnosis
Li Jinxiu [35]	2019	Ischemic	152	60–80	IFA	Not specified	Analyzed PCT and hs-CRP values in SAP diagnosis	Increased PCT and hs-CRP in SAP patients

Among the biomarkers investigated, only PCT had a sufficient number and quality of studies for meta-analysis. Other biomarkers, including CRP and IL-6, were reported in the literature but were excluded due to limited available studies. CRP and IL-6 were reported to be elevated in SAP patients in several studies [20–22]. Additional inflammatory markers such as TNF-α and IL-1β) were mentioned in a small number of studies [23, 24].

The quality of the included studies was evaluated using the Cochrane Risk of Bias Tool (Fig. 2B). Domains assessed included random sequence generation, allocation concealment, blinding, and outcome assessment. Studies were classified as having low, high, or unclear risk of bias based on methodological details. Studies lacking adequate randomization or allocation concealment were marked as high risk. Unclear risk was assigned when methodological details were insufficient to determine bias.

### The diagnostic value of elevated PCT levels in SAP

**Fig. 3:**
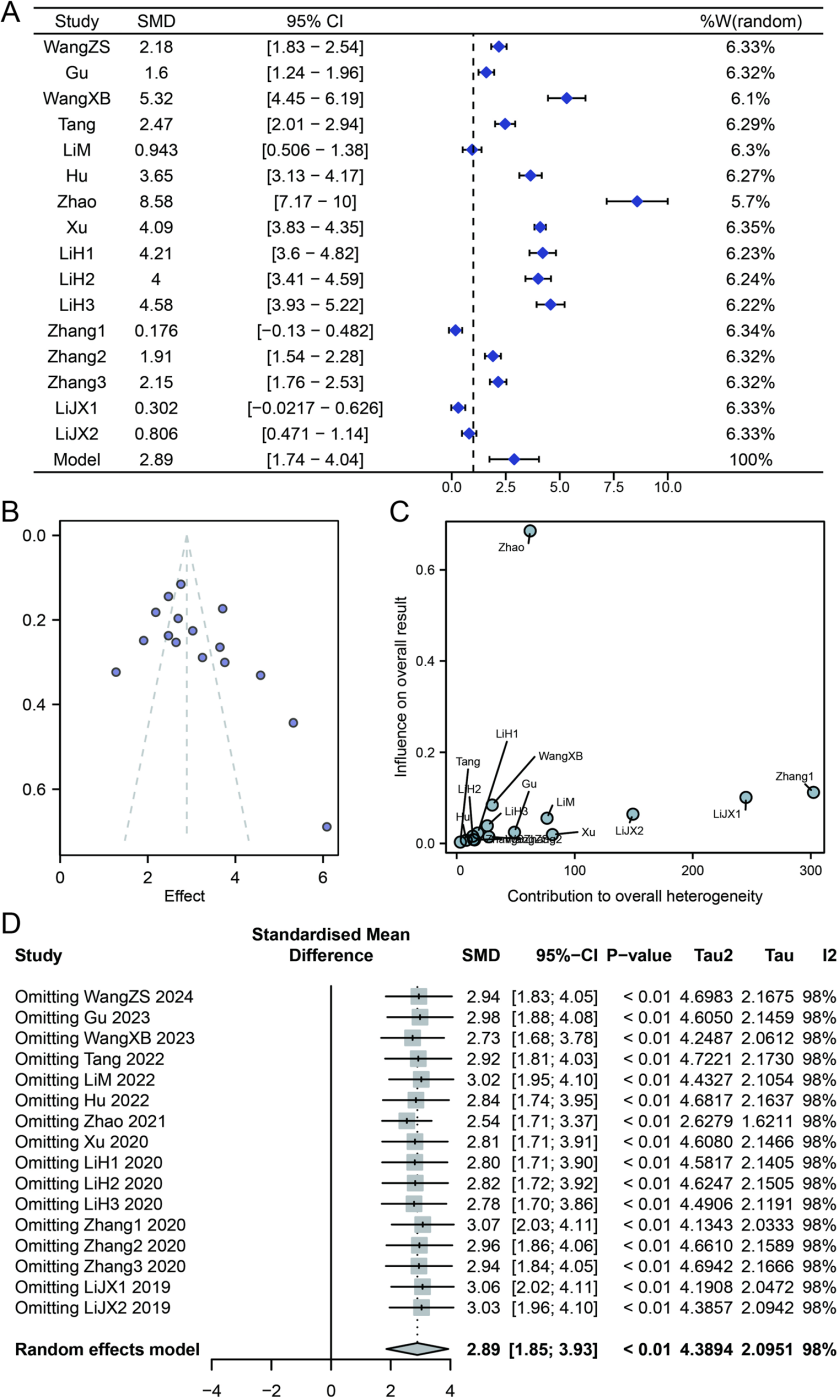
Meta-analysis of procalcitonin (PCT) levels in patients with stroke-associated pneumonia (SAP). Note: (**A**) Forest plot comparing PCT levels between SAP and non-SAP groups using a random-effects model (tau² = 4.4, I² = 98.33%); (**B**) Funnel plot assessing publication bias. Each dot represents a study, with the x-axis indicating the estimated effect size and the y-axis representing the standard error. Asymmetry may suggest potential publication bias, such as the absence of small studies or those with nonsignificant effects; (**C**) Baujat plot identifying sources of heterogeneity. The x-axis shows each study’s contribution to overall heterogeneity (Q statistic), while the y-axis reflects its influence on the pooled effect estimate; (**D**) Sensitivity analysis plot showing the stability of pooled standardized mean difference (SMD) after sequential exclusion of each study

**Fig. 4:**
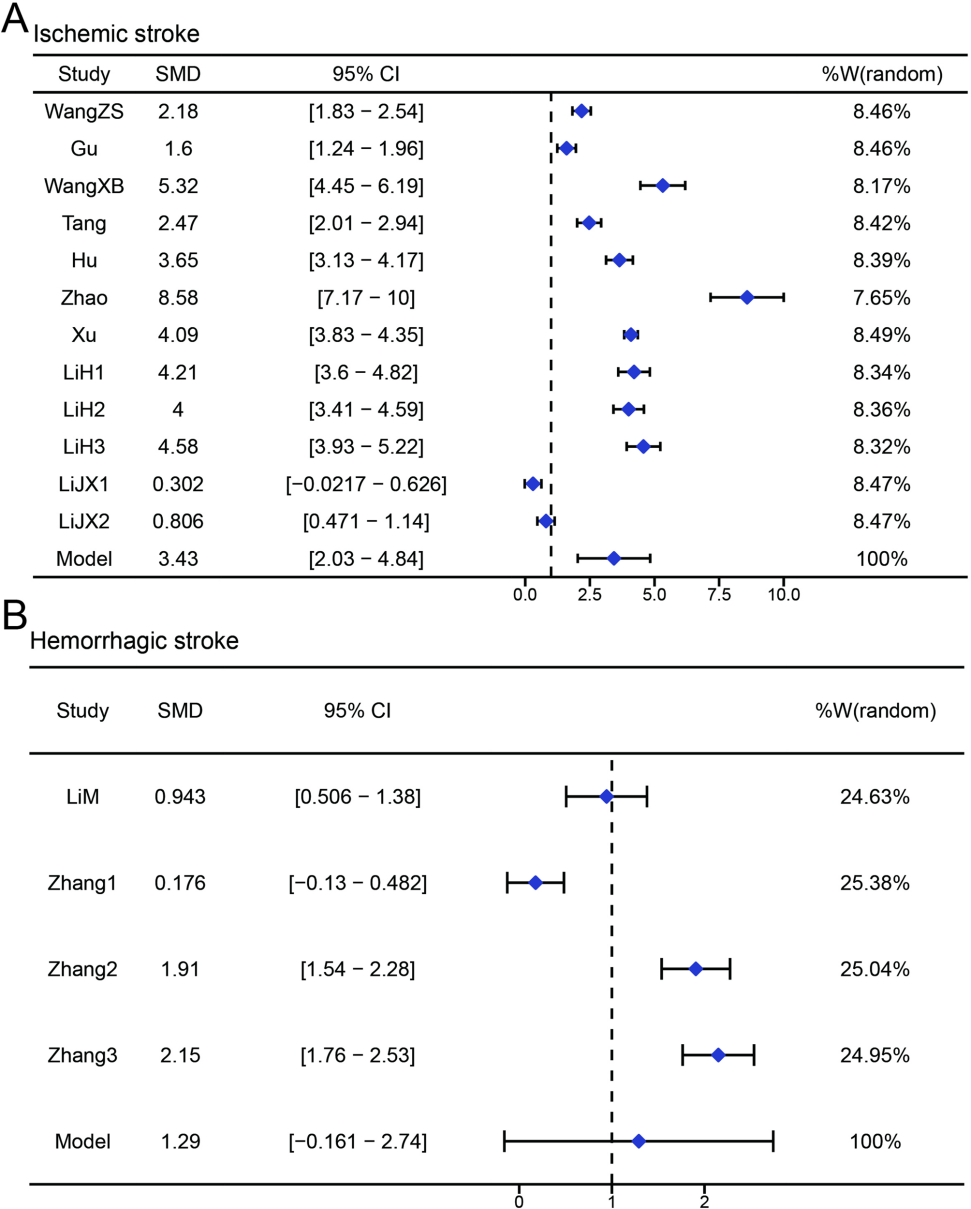
Subgroup meta-analysis of procalcitonin (PCT) levels in different types of stroke-associated pneumonia (SAP). (**A**) Forest plot of PCT levels in patients with ischemic stroke-associated pneumonia; (**B**) Forest plot of PCT levels in patients with hemorrhagic stroke-associated pneumonia

As shown in Fig. 3A, a meta-analysis was conducted using a random-effects model (tau² = 4.4, I² = 98.33%). Pooled results showed that procalcitonin (PCT) levels were higher in patients with stroke-associated pneumonia (SAP) compared to non-SAP controls, with a standardized mean difference (SMD) of 2.89 and a 95% confidence interval (CI) of [1.74–4.04]. Publication bias was assessed using funnel and Baujat plots (Fig. 3B -C). The funnel plot showed an approximately symmetrical distribution of effect sizes versus standard errors. In the Baujat plot, most studies contributed minimally to heterogeneity and effect size. Three studies (Zhao, LiJX, Zhang) demonstrated higher influence on heterogeneity. Sensitivity analysis (Fig. 3D) was performed by sequentially excluding individual studies. The pooled SMD and 95% CI remained stable across iterations, with I^2^ consistently at 100%. Subgroup analysis based on stroke type was also conducted. In ischemic stroke patients, the pooled SMD was 3.43 (95% CI = [2.03–4.84], Fig. 4A). In hemorrhagic stroke patients, the SMD was 1.29 (95% CI = [–0.16–2.74], Fig. 4B).

### Progress in research on clinical immune and inflammatory biomarkers and predictive models for SAP

**Fig. 5:**
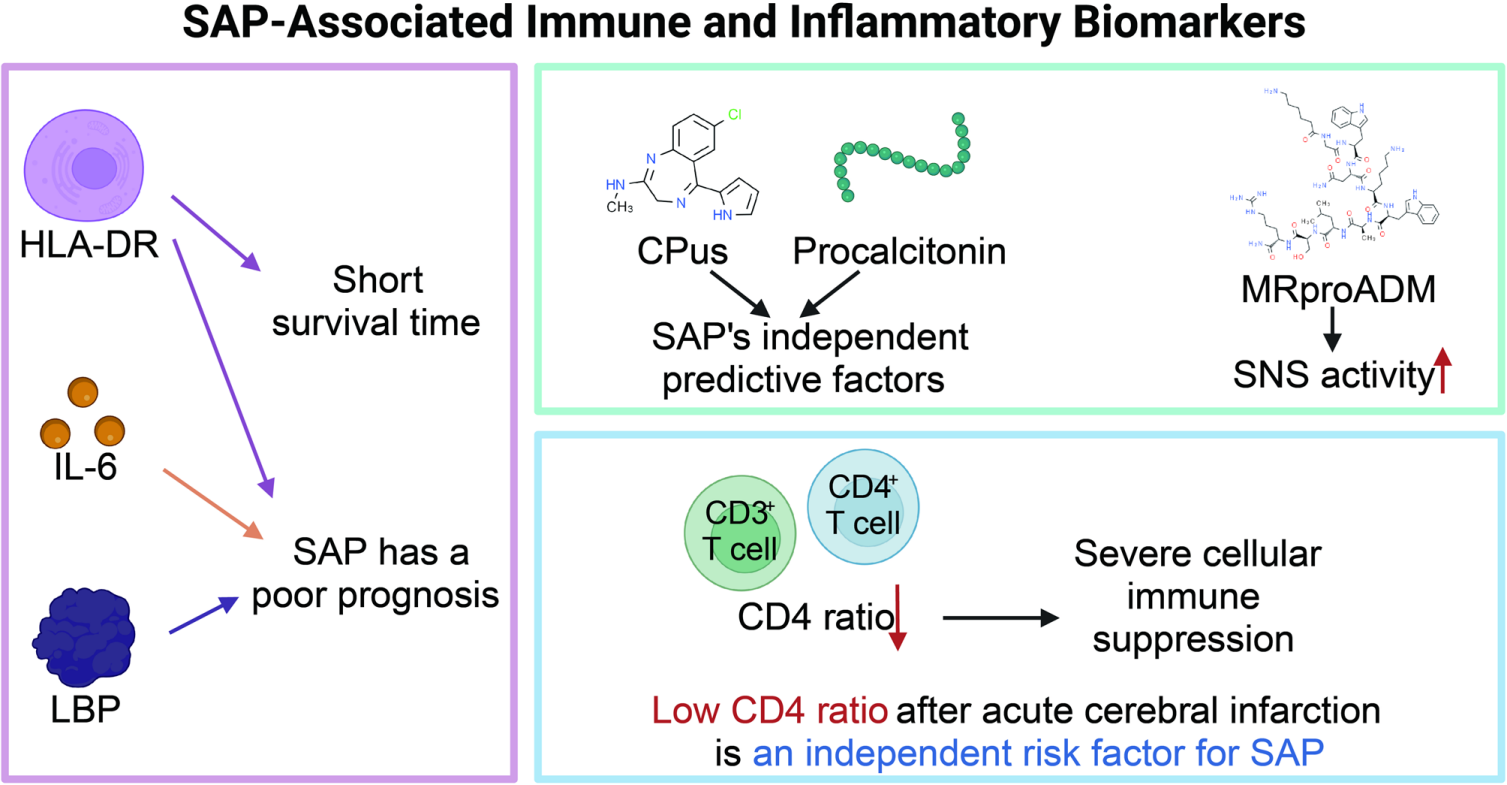
Immune and inflammatory biomarkers associated with SAP

This study found that various clinical immune and inflammatory biomarkers are significantly associated with the occurrence and prognosis of SAP (Fig. 5).

Among monocytic markers, reduced expression of mHLA-DR and elevated levels of IL-6 and LBP were related to poor outcomes in stroke patients. Serum biomarkers such as high-sensitivity copeptin (CPus), high-sensitivity procalcitonin (PCTus), and mid-regional pro-adrenomedullin (MRproADM) were strongly associated with SAP and unfavorable post-stroke outcomes, with CPus and PCTus identified as independent predictors of SAP. Inflammatory indicators, including the systemic immune-inflammation index (SII), neutrophil-to-lymphocyte ratio (NLR), and neutrophil percentage-to-albumin ratio (NPAR), also demonstrated predictive value for SAP risk, particularly NLR, which was significantly elevated within 24 h after ischemic stroke and correlated with pneumonia severity. PCT, with higher infection specificity, was effective for SAP diagnosis.

Recent studies have also identified novel biomarkers such as brain-derived neurotrophic factor (BDNF) and circulating microRNAs, while the application of machine learning-based prediction models has further improved SAP risk stratification. The immunomodulator IL-37 has shown potential in reducing SAP incidence (Table S1).

### Evaluation and application of clinical prediction models for SAP

**Fig. 6:**
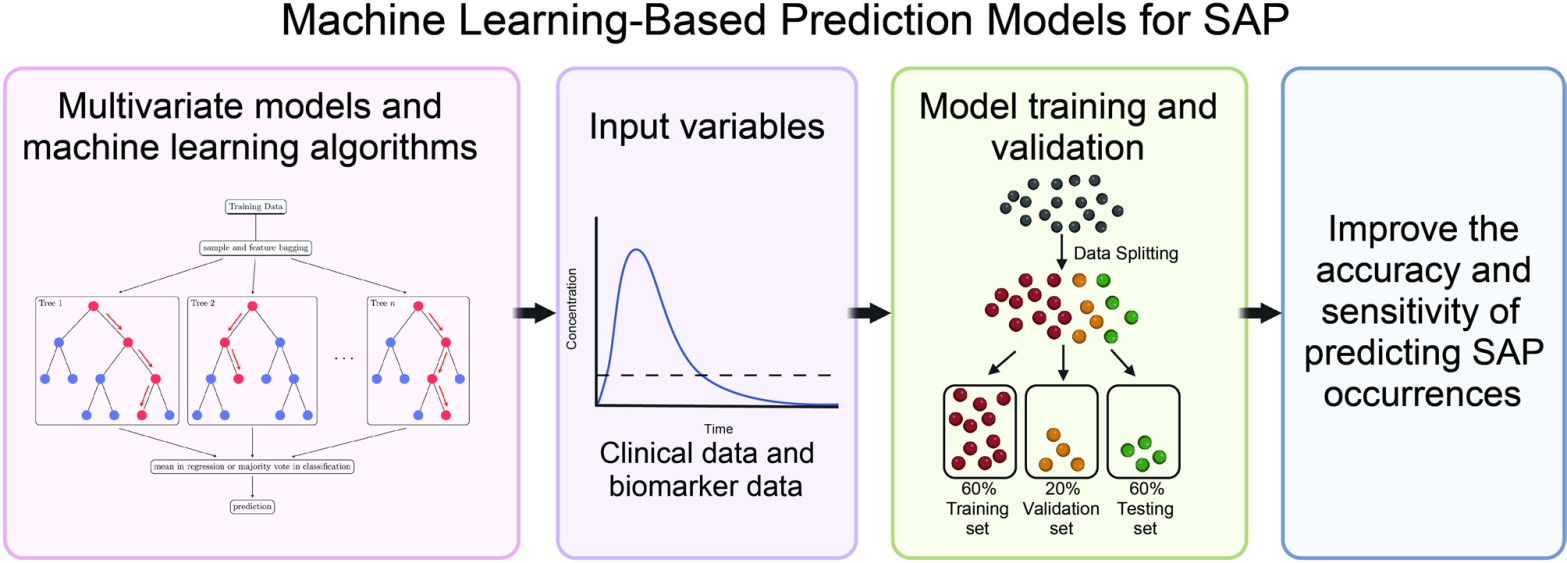
Mechanism diagram of machine learning prediction models

Several prediction models based on common post-stroke clinical variables have been developed to assess the risk of SAP (Fig. 6; Tables 2 and 3).

**Table 2:** Biomarkers of immunity, inflammation, and stress in SAP and post-stroke infection

Biomarkers	Sample	Direction	Disease	Stroke stage	Main findings	PMID
mHLA-DR	Plasma	Immunity	SAP	Day 1	Down	35,509,104, 35,748,095
IL-6	Plasma	Inflammation	SAP	Day 1	Up	35,509,104, 35,748,095
LBP	Plasma	Inflammation	SAP	Day 1	Up	35,509,104, 35,748,095
CPus	Serum	Stress	SAP	First 4 days	Up	25,612,858
PCTus	Serum	Inflammation	SAP	First 4 days	Up	25,612,858
CTproET	Serum	Stress	SAP	First 4 days	Up	25,612,858
MRproANP	Serum	Stress	SAP	First 4 days	Up	26,343,840, 25,612,858
MRproADM	Serum	Inflammation	SAP	First 4 days	Up	25,612,858, 28,484,421
suPAR	Serum	Immunity	SAP	24 h	Up	28,484,421
SAA	Serum	Immunity	SAP	48 h	Up	28,484,421
CD3 + T cells	Whole blood	Immunity	SAP	Day 1	Down	26,343,837
CD4 + T cells	Whole blood	Immunity	SAP	Day 1	Down	26,343,837
CD4:CD8 ratio	Whole blood	Immunity	SAP	Day 1	Down	26,343,837
NLR	Plasma	Inflammation	SAP	Day 1	Up	29,355,906
CRP	Serum	Inflammation	SAP	Day 1	Up	33,873,077, 26,742,037
PCT	Serum	Inflammation	Post-stroke infection	Day 1 and day 4	Up	29,330,443, 34,540,122
Copeptin	Plasma	Stress	Post-stroke infection	72 h	Up	26,343,840
LCN2	Plasma	Immunity	Post-stroke infection	1 week	Up	26,584,429
Tim-4	Plasma	Inflammation	Post-stroke infection	Day 2 and day 5	Up	37,875,813
PA	Serum	Oxidative stress	Post-stroke infection	Day 1	Down	37,795,380
SOD	Serum	Oxidative stress	Post-stroke infection	Day 1	Up	33,240,188
IL-10	Plasma	Inflammation	Post-stroke infection	First 4 days	Up	34,540,122

Note: SAP: Stroke associated pneumonia; mHLA-DR: monocytic HLA-DR; IL-6: Interleukin-6; LBP: lipopolysaccharide-binding protein; CPus: Ultrasensitive copeptin; PCTus: Ultrasensitive procalcitonin; CTproET: C-terminalpro-endothelin-1; MR-proANP: Midregional pro-atrial natriuretic peptide; MR-proADM: Midregionalpro-adrenomedullin; suPAR: Soluble urokinase-type plasminogen activator receptor; SAA: Serum amyloid A protein; NLR: Neutrophil-to-Lymphocyte Ratio; CRP: C-reactive protein; PCT: Procalcitonin; LCN2: Lipocalin-2; Tim-4: T cells Immunoglobulin and mucin domain-4; PA: Prealbumin; SOD: Serum superoxide dismutase; IL-10: Interleukin-10

**Table 3:** Components of SAP prediction models

Name	VHA Score [36]	A^2^DS^2^ score [37]	PANTHERIS score [38]	AIS-APS score [39]	ICH-APS score [40]		ISAN score [41]	ACDD^4^ score [42]
					ICH-APS-A	ICH-APS-B		
Stroke location and type	IS	IS	IS of MAC	AIS	ICH without hematoma	ICH	AIS and ICH	AIS and ICH
Items								
Age	√	√	√	√	√	√	√	√
Sex		√					√	
RR_syst_ > 200mmHg*			√					
Current smoking				√	√	√		
Excess alcohol consumption					√	√		
NIHSS score	√	√		√	√	√	√	
Pre-stroke dependence (mRS)				√	√	√	√	
Dysphagia	√	√		√	√	√		√
Dysarthria								√
Found down at symptom onset	√							
OCSP subtype				√				
Intraventricular extension					√	√		
Extension into ventricles					√			
Hematoma volume						√		
GCS score			√	√	√	√		
WBC			√					
Atrial fibrillation		√		√				
CHF				√				√
COPD				√	√	√		
Previous pneumonia	√							
Admission glucose				√				

Note: VHA: Veteran’s Health Administration Cohort; A^2^DS^2^: Age, Atrial Fibrillation, Dysphagia Sex, Severity; PANTHERIS: Preventive Antibacterial Therapy in Acute Ischemic Stroke; AIS-APS: Acute Ischemic Stroke-Associated Pneumonia Score; ICH-APS: Intracerebral haemorrhage-Associated Pneumonia Score; ISAN: Pre-stroke Independence (modified Rankin scale), Sex, Age, National Institutes of Health Stroke Scale; ACDD^4^: Age, Congestive heart failure, Dysarthria, Dysphagia; IS: ischemic stroke; AIS: Acute ischemic stroke; MAC: Middle cerebral artery; ICH: Intracerebral haemorrhage; R_Rsyst_: systolic arterial blood pressure; NIHSS: National Institutes of Health Stroke Scale; mRS: modified Rankin scale; OCSP: Oxfordshire Community Stroke Project; GCS: Glasgow coma scale; WBC: White blood cell; CHF: Congestive heart failure; COPD: Chronic obstructive pulmonary disease. *Measured within the first 24 h after admission

Common independent risk factors include age, NIHSS score, and dysphagia. Widely used SAP prediction models include the VHA score, A2DS2 score, PANTHERS score, AIS-APS score, ICH-APS score (including ICH-APS-A and ICH-APS-B), ISAN score, ACDD4 score, and ICH-LR2S2 score. External validation studies suggest that the A2DS2 score offers high sensitivity for early screening, whereas the AIS-APS score provides greater specificity and is more suitable for risk stratification in acute ischemic stroke.

Recent studies have also employed machine learning and natural language processing approaches to construct new predictive models, which integrate clinical and immune biomarker data to improve predictive accuracy. However, several models were not specifically designed for SAP or lacked sufficient external validation (Table S2).

### The efficacy and challenges of prophylactic antibiotics in SAP treatment

Several studies have evaluated the effectiveness of prophylactic antibiotics in the prevention and management of SAP. While there is general consensus that antibiotic treatment should be initiated promptly upon confirmed SAP diagnosis, the optimal timing and choice of antibiotics remain unclear.

Multiple randomized controlled trials have shown limited efficacy of prophylactic antibiotic use in reducing SAP incidence or improving long-term functional outcomes, and current clinical guidelines do not recommend routine prophylactic use. Some evidence suggests that prophylactic antibiotics may be more effective in preventing urinary tract infections than pneumonia. The efficacy of antibiotic treatment is further influenced by factors such as pathogen complexity, diagnostic challenges, and impacts on gut microbiota. Although broad-spectrum antibiotics offer wide microbial coverage, their use raises concerns about antibiotic resistance (Table S3).

### The application and challenges of clinical care and immunomodulation in SAP prevention

Studies have shown that improving nursing quality, enhancing oral hygiene, and conducting early swallowing assessments can reduce the risk of SAP in stroke patients.

Some evidence supports the use of cilostazol in tube-fed patients as a preventive measure. Malnutrition is considered a risk factor for SAP, and vitamin supplementation may serve as a potential adjunctive therapy. Although immunomodulatory therapy has been proposed as a novel intervention, there is currently no consensus regarding its therapeutic targets or effectiveness. Further clinical studies are needed to clarify the underlying mechanisms and evaluate the efficacy of such interventions (Table S4).

## Discussion

This study comprehensively evaluates the clinical immunology and inflammatory biomarkers, predictive models, strategies for prophylactic antibiotic use, and nursing and immune modulation measures for SAP through a systematic review and meta-analysis.

The findings reveal that the occurrence of SAP is closely associated with various clinical immunological and inflammatory biomarkers. Biomarkers such as HLA-DR, IL-6, and PCT have been widely applied in the early diagnosis and risk prediction of SAP [43, 44]. Notably, elevated PCT levels are significantly associated with the occurrence of SAP, underscoring the importance of PCT in the diagnosis and monitoring of SAP [45, 46]. However, due to the high heterogeneity among studies, further research is needed to explore the application of these biomarkers across different populations and clinical contexts to optimize their diagnostic efficacy for SAP.

Various clinical prediction models have been developed for the early identification of high-risk populations, including the VHA score, A2DS2 score, and AIS-APS score [46–48]. These models combine clinical symptoms, signs, and relevant examinations of stroke patients to provide accurate risk assessment tools. However, there are still some issues in their practical application, such as the lack of a specific design for SAP during the model development process and incomplete data collection. Additionally, with the advancement of computer technology, the application of machine learning and NLP techniques in predictive models has shown promising prospects [50, 51]. Future research should continue to optimize these models, particularly by incorporating immune markers to enhance their predictive performance.

The use of prophylactic antibiotics in the prevention and treatment of SAP remains highly controversial. Although some studies support the use of prophylactic antibiotics [52], large-scale clinical trials have not demonstrated a significant reduction in the incidence and mortality rates of SAP [53, 54]. Potential reasons include the complex pathogenesis of SAP, multiple pathogen pathways, and inadequate antibiotic coverage. Additionally, the effectiveness of different types of antibiotics varies, with broad-spectrum antibiotics potentially leading to the development of resistant strains [52]. Future research should further investigate the impact of various antibiotic types and administration strategies on SAP to develop more scientific and personalized treatment plans.

In patients with CAP and carotid atherosclerosis, elevated NLR reflects a systemic inflammatory state, a broad immune response triggered by infection, trauma, or other stressors. Unlike localized immune responses, systemic inflammation affects overall immune function, altering the number and activity of various cell types, including neutrophils and lymphocytes [55, 56]. SAP patients often have comorbidities such as COPD, diabetes, and hypertension, which exacerbate systemic inflammation and may lead to immune dysregulation [8]. Bacterial infections in SAP have been well-documented, with studies indicating a significantly increased risk of bacterial pneumonia, particularly in stroke patients with dysphagia or impaired consciousness. Strong associations between SAP and bacterial infections, including Gram-negative bacilli, have been reported [57, 58].

High-quality nursing and scientific immune modulation play crucial roles in the prevention of SAP. Studies have shown that enhanced oral hygiene care, early screening for swallowing difficulties, and appropriate feeding methods can effectively reduce the incidence of SAP [36, 59–61]. Additionally, supplementation with nutrients such as vitamin D and vitamin E has been shown to have adjunctive therapeutic effects for SAP patients [39, 40]. However, the specific targets, pathways, and duration of immune modulation therapy remain unclear, presenting challenges for its clinical application [41, 42]. Future research needs to further elucidate the mechanisms of immune modulation and design feasible clinical trials to verify its efficacy.

This study systematically evaluated the clinical utility of PCT in the early diagnosis of SAP through a comprehensive meta-analysis. The results demonstrated that PCT levels were significantly elevated in SAP patients and exhibited favorable specificity and sensitivity. Sensitivity analysis and publication bias assessment further supported the stability and reliability of PCT as a biomarker. Compared to conventional indicators such as the NLR, PCT showed superior diagnostic performance in the context of bacterial infection.

Incorporating PCT into traditional prediction models, such as A2DS2 and AIS-APS, may improve their diagnostic accuracy. Although the A2DS2 score is simple and practical, it lacks consideration of inflammatory or infectious markers, which limits its sensitivity. The AIS-APS model focuses on pneumonia risk post-stroke but does not integrate updated biomarker data. This study highlights that combining PCT and other clinical variables may contribute to the development of more accurate SAP prediction models. Additionally, risk stratification based on PCT levels (< 0.25 ng/mL for low risk, 0.25–0.5 ng/mL for intermediate risk, and > 0.5 ng/mL for high risk) may support clinical decision-making and aid in building individualized early warning systems. NLR, a widely accessible and low-cost systemic inflammatory indicator, has been associated with SAP and other infectious disease outcomes [4, 6, 62]. Although NLR cannot directly distinguish bacterial infections, its combination with PCT may offer a more comprehensive risk assessment framework, enhancing the identification of high-risk patients and optimizing the timing of clinical interventions.

This study has certain limitations. First, heterogeneity in study design, sample characteristics, and definitions of biomarker thresholds may affect the consistency and robustness of the meta-analysis results. Some studies had a high risk of selection bias, which may reduce the reliability of the findings for clinical application. Additionally, inconsistent PCT threshold definitions across studies limit the generalizability of the pooled results. Finally, the absence of complete raw data in several studies restricted further meta-regression analysis and subgroup exploration.

Future research should focus on conducting large-scale, multicenter prospective studies to standardize biomarker measurement methods and threshold definitions. It is also important to integrate immune-inflammatory biomarkers such as PCT into AI-based prediction models to improve clinical applicability and diagnostic accuracy. High-quality randomized controlled trials are needed to evaluate the long-term effects of various antibiotic regimens and immunomodulatory strategies in the prevention and treatment of SAP. In addition, further studies are required to validate the effectiveness of nursing interventions and nutritional support in reducing SAP risk. A multidimensional prediction and management strategy supported by high-quality evidence is essential to enhance early identification and improve clinical outcomes in patients with SAP.

## Conclusions

This meta-analysis provides evidence that PCT is significantly elevated in patients with SAP and demonstrates favorable diagnostic accuracy. The findings support the potential role of PCT as a valuable biomarker for early identification of SAP, particularly when combined with clinical symptoms and other inflammatory markers. Incorporating PCT into existing clinical prediction models may improve risk stratification and guide early intervention strategies. However, variability in study quality and biomarker thresholds highlights the need for further research. Future studies should focus on validating standardized biomarker criteria and integrating novel markers into predictive frameworks to enhance clinical decision-making in SAP management.

## Electronic supplementary material

Below is the link to the electronic supplementary material.

Supplementary Material 1

## Data Availability

The datasets used and analysed during the current study are available from the corresponding author on reasonable request.

## Abbreviations

AIS: Acute Ischemic Stroke
BDNF: Brain-Derived Neurotrophic Factor
CRP: C-Reactive Protein
hs-CRP/Albumin Ratio: High-sensitivity C-reactive Protein/Albumin Ratio
IL-1β: Interleukin-1 beta
IL-6: Interleukin-6
IS: Ischemic Stroke
LCN2: Lipocalin-2
miRNA: microRNA
NLP: Natural Language Processing
NIHSS: National Institutes of Health Stroke Scale
PA: Serum prealbumin
PCT: Procalcitonin
PCTus: High-sensitivity Procalcitonin
RCTs: randomized controlled trials
NLR: Neutrophil-to-lymphocyte ratio
SAA: Serum Amyloid A
SAP: Stroke-Associated Pneumonia
SIS: Systemic Immunosuppression
SOD: Superoxide dismutase
Tim-4: T-cell immunoglobulin and mucin domain-4
TNF-α: Tumor Necrosis Factor-alpha
UTIs: urinary tract infections
VHA: Veterans Health Administration
