# Supplementary material for: Procalcitonin and biomarkers for stroke-associated pneumonia: a systematic review and meta-analysis
Source: BMC Pulm Med. 2025 Jun 9;25:286. doi: 10.1186/s12890-025-03750-6 (PMC12147346; doi:10.1186/s12890-025-03750-6)
Supplement: Supplementary file 1 — Supplementary Material 1 [file 12890_2025_3750_MOESM1_ESM.docx]

**Table S1. Overview of Clinical Immune and Inflammatory Biomarkers Associated with Stroke-Associated Pneumonia.**

| **Biomarker** | **Sample Type** | **Trend** | **Association with SAP or outcomes** | **References (PMID)** |
| --- | --- | --- | --- | --- |
| mHLA-DR | Plasma | ↓ | Low levels linked to poor stroke prognosis | 27733675; 31269910 |
| IL-6 | Plasma | ↑ | Elevated levels associated with poor stroke outcomes, SAP | 27733675; 31269910 |
| LBP | Plasma | ↑ | Elevated in SAP; related to inflammation and infection risk | 27733675; 31269910 |
| CPus (High-sensitivity Copeptin) | Serum | ↑ | Independent predictor of SAP; linked to 3-month outcomes | 22710977; 32098866 |
| PCTus (High-sensitivity Procalcitonin) | Serum | ↑ | Independent predictor for SAP; related to infection risk and outcomes | 22710977; 32098866 |
| MRproADM | Serum | ↑ | Linked to SAP occurrence and pneumonia severity | 31365180; 22710977 |
| suPAR | Serum | ↑ | Associated with pneumonia occurrence after stroke | 31365180 |
| SAA | Serum | ↑ | Elevated in SAP; involved in inflammatory cell recruitment | 31365180 |
| SII Index) | Blood | ↑ | Independent risk factor for SAP | 37938870 |
| CD4:CD8 Ratio | Whole blood | ↓ | Lower ratio independently associated with SAP | 30662678 |
| NLR | Plasma | ↑ | Elevated 24h post-stroke; predicts SAP severity and incidence | 29967014; 37720840 |
| NPAR | Plasma | ↑ | Superior predictive value for SAP compared to NLR | 37756038 |
| CRP | Serum | ↑ | Linked to SAP severity; limited specificity due to confounders | 30196790 |
| PCT | Serum | ↑ | Specific biomarker for infection post-stroke; effective for SAP diagnosis | 20052623; 33389464 |
| Eosinophil Count | Blood | ↓ | Lower counts associated with increased SAP risk | 35677203 |
| LCN2 | Plasma | ↑ | Elevated levels associated with higher risk of infection post-stroke | 27152948 |
| Tim-4 | Monocytes | ↑ | Elevated expression linked to higher infection risk post-stroke | 29233585 |
| PA | Serum | ↓ | Lower levels correlated with increased infection risk post-stroke | 27476523 |
| SOD | Serum | ↑ | Elevated levels associated with SAP risk | 31696761 |
| BDNF | Serum | ↓ | Lower levels associated with SAP and poor stroke outcomes | 35287688; 27815836 |
| miRNA (miR-21) | Blood | Altered | Altered levels linked to post-stroke pneumonia risk | 33712063 |
| Machine-learning prediction models | Integrated biomarkers | Improved Accuracy | Enhanced SAP predictive accuracy combining clinical data and biomarkers | 38063586 |
| IL-37 (Immunomodulator) | Intervention | ↓ | Potential for reducing SAP incidence | 34092245 |

Note: ↑ Increased levels; ↓ Decreased levels. mHLA-DR, Monocytic human leukocyte antigen-DR; IL-6, Interleukin-6; LBP, Lipopolysaccharide-binding protein; MRproADM, Mid-regional pro-adrenomedullin; suPAR, Soluble urokinase-type plasminogen activator receptor; SAA, Serum amyloid A; NLR, Neutrophil-to-lymphocyte ratio; NPAR, Neutrophil percentage-to-albumin ratio; CRP, C-reactive protein; LCN2, Lipocalin-2; PA, Prealbumin; SOD, Superoxide dismutase; BDNF, Brain-derived neurotrophic factor; miRNA, microRNA.

**Table S2. Components of SAP Prediction Models and References**

| SAP Prediction Model | Stroke Type | Major Components Included | References (PMID) |
| --- | --- | --- | --- |
| VHA Score | IS | Age, NIHSS score, Dysphagia, Found down, Previous pneumonia | 20197702 |
| A2DS2 Score | IS | Age, Atrial fibrillation, Dysphagia, Sex, NIHSS | 22798325 |
| PANTHERIS Score | IS (MAC) | Age, Hypertension, Smoking, COPD, NIHSS | 23461541 |
| AIS-APS Score | AIS | Age, NIHSS, Dysphagia, Atrial fibrillation, Smoking, Admission glucose | 23482598 |
| ICH-APS-A Score | ICH | Age, NIHSS, Dysphagia, GCS, Intraventricular extension | 25028448 |
| ICH-APS-B Score | ICH | Age, NIHSS, GCS, Intraventricular extension, Hematoma volume | 35530955 |
| ISAN Score | AIS and ICH | Age, Sex, NIHSS, Pre-stroke dependence, Dysphagia | 38249863 |
| ACDD4 Score | AIS and ICH | Age, Congestive heart failure, Dysarthria, Dysphagia | 27823836 |
| ICH-LR2S2 Score | ICH | NIHSS, Age, Dysphagia, Hematoma characteristics | 35509104 |

Note：IS: ischemic stroke; AIS: acute ischemic stroke; ICH: intracerebral hemorrhage; MAC: middle cerebral artery; NIHSS: National Institutes of Health Stroke Scale; COPD: Chronic obstructive pulmonary disease; GCS: Glasgow coma scale

**Table S3. Summary of Studies on Preventive Antibiotics in SAP**

| **Study Type** | **Intervention** | **Main Outcome on SAP** | **Other Findings** | **References (PMID)** |
| --- | --- | --- | --- | --- |
| Systematic review | Antibiotics post-diagnosis | Recommended immediate use post-SAP diagnosis | Optimal timing and agent uncertain | 31903430 |
| RCT (multicenter) | Preventive antibiotics | No significant reduction in SAP incidence | No improvement in 3-month outcomes | 26343840; 25612858; 28484421 |
| RCT | Preventive antibiotics | Did not prevent SAP | Reduced UTI incidence | 26343837; 29355906 |
| Cohort study | Antibiotics in SAP | Effectiveness influenced by pathogen complexity | Diagnosis challenges highlighted | 33873077; 26742037; 29330443 |
| Comparative analysis | Broad-spectrum antibiotics (e.g., ceftriaxone, quinolones) | Broad coverage observed | Resistance risk; microbiome impact | 32257701; 26343840; 33873077 |

**Table S4. Summary of Nursing, Nutritional, and Immunological Interventions in SAP.**

| **Intervention Type** | **Strategy/Agent** | **Main Findings** | **References (PMID)** |
| --- | --- | --- | --- |
| Nursing quality | Overall care improvement | Associated with reduced mortality and better outcomes | 34540122 |
| Oral hygiene | Oral care, reduced colonization | Reduced SAP incidence; colonization is key risk | 26584429; 37875813 |
| Oral hygiene & feeding | Early swallow screening, feeding method | Improved oral health; decreased SAP incidence | 37795380; 33240188; 33124506; 34269936 |
| Pharmacologic support | Cilostazol in tube-fed patients | Reduced SAP risk in enteral feeding context | 29680945 |
| Nutritional support | Vitamin D and E supplementation | Potential as adjunct therapy; malnutrition is a risk | 37537542; 32021127; 32910689 |
| Immunotherapy | Modulation of stroke-induced immunosuppression | Feasibility discussed; efficacy unclear | 31447768; 31697040 |
| Experimental agent | Dexmedetomidine | Did not improve SAP or reduce infarct volume in animals | 37601052 |
